# Interspecific hybridization history of *Vaccinium* berry crops and potential in wild relatives

**DOI:** 10.1093/hr/uhaf246

**Published:** 2025-09-17

**Authors:** Anisa M Khalid, Juliana Benevenuto, Paul M Lyrene, Patricio R Munoz

**Affiliations:** Horticultural Sciences Department, University of Florida, Gainesville, FL 32611, USA; Horticultural Sciences Department, University of Florida, Gainesville, FL 32611, USA; Horticultural Sciences Department, University of Florida, Gainesville, FL 32611, USA; Horticultural Sciences Department, University of Florida, Gainesville, FL 32611, USA

## Abstract

Wild species have been extensively used as a reservoir of genetic variability in plant breeding. Both blueberries and cranberries are crops from the highly diverse *Vaccinium* genus that have benefited from interspecific hybridizations throughout their domestication history. In this review, we compiled all documented interspecific hybridizations performed for blueberry and cranberry aiming to guide future breeding efforts. We report the traits of interest, success and failure of crosses, and give the taxonomic sections of the species involved. Out of the 500 species listed in *Vaccinium*, only 42 have been tested for hybridization so far. Successful crosses with fertile progenies have been reported across distantly related sections. Considering the polyphyletic nature of *Vaccinium*, the definition of crop wild relatives for these crops could be expanded to incorporate other genera. This review highlights the enormous potential of the wild gene pools for breeding of *Vaccinium* berry crops, and the need to characterize these species and establish germplasm collections to face the agricultural challenges ahead.

## Introduction

The genus *Vaccinium* is renowned for economically important crops, particularly blueberries (*Vaccinium* spp. sect. *Cyanococcus*) and cranberries (*Vaccinium macrocarpon* sect. *Oxycoccus*). Part of the Ericaceae family, subfamily Vaccinoideae, and tribe Vaccinieae, the genus is comprised of ~500 species across 29 taxonomic sections, each with diverse foliage, flowers, and fruits, offering vast potential for horticultural and ornamental use [1, 2]. Coveted for their high nutritional value, *Vaccinium* crops are becoming increasingly demanded in the global market, and therefore, require consistent genetic improvement to meet ongoing production and quality requirements [3, 4].

Historically, breeding programs for *Vaccinium* crops have used the genetic diversity of wild relatives to enhance breeding populations. A notable example was an interspecific hybridization of the Northern Highbush Blueberry (NHB), with primarily *Vaccinium corymbosum* background, and the Florida native *Vaccinium darrowii* [5–7]. The incorporation of low-chill requirement from *V. darrowii* enabled the hybrids to thrive in diverse climates and created a novel industry of Southern Highbush Blueberry (SHB), transforming blueberries into a now global crop [8]. This example underscores the value of evaluating wild *Vaccinium* species for crop improvement.

The complex taxonomy of *Vaccinium* convolutes the definition of blueberry and cranberry’s true Crop Wild Relatives (CWRs). Currently, the CWR definition arbitrarily encompasses just *Vaccinium* species for both blueberry and cranberry, but the known polyphyly of the *Vaccinium* genus has crucial implications for potential interspecific hybridizations, possibly resulting in an underestimate of available diversity for breeding efforts [9, 10].

Despite the deployment of interspecific hybrids for improvement of *Vaccinium* berry crops for over a century, a comprehensive list of species used for breeding and their outcomes remains outdated in the literature. The most recent review on interspecific hybridization in *Vaccinium* dates to 2009 [11], leaving a gap in our knowledge of subsequent advancements. Additionally, unlike previous reviews that took a descriptive approach, this review aims to systematically explore the history of wild species introgression in *Vaccinium* crop improvement through objective query-based screening of the literature. Furthermore, we synthesize the information gathered from the literature with novel phylogenetic findings to draw conclusions about the future potential hybridizations.

With the rapid development of blueberry and, to a lesser extent, cranberry globalization, an increasing number of highly adaptable cultivars with novel traits are urgently needed [12]. However, while there are many well-documented sources of the use of wild species, there are still major problems to overcome such as the fragmented information about interspecific hybridization throughout history, incomplete understanding of the true CWR, and lack of characterization for most wild relatives, especially those in the tropics. To address these gaps, we set two main objectives for this review: (i) to compile all artificial interspecific hybridizations documented for *Vaccinium* crop breeding, highlighting both successes and failures, and (ii) to leverage botanical knowledge, including phylogenetic relationships, to refine the CWR definition for blueberry and cranberry. Notably, this review will not only assess past progress but also highlight the challenges and future potential of wild species in *Vaccinium* breeding.

## Interspecific hybridization in *Vaccinium* crops

The *Vaccinium* genus is home to many economically important species. These range from purely wild species, managed wild species, to domesticated crops grown commercially. In this section we will discuss mostly the domesticated groups as breeding efforts have involved interspecific hybridization. Studying interspecific and intersectional hybridization can contribute to our knowledge of the primary, secondary, and tertiary gene pools. This historical perspective can also guide future breeding efforts by highlighting successes and failures across the phylogenetic tree.

Interspecific hybridization of *Vaccinium* species is possible due to the same base number of chromosomes (*n* = 12) and high genomic collinearity [8]. Ploidy levels, however, are various across the genus, with natural occurrences of diploids, tetraploids, and hexaploids, sometimes within the same species. Some species or individuals are also known to produce a high frequency of unreduced (2*n*) gametes that facillitate crossing across differing ploidy levels [13, 14]. An effective artificial method that has been used to overcome this crossing barrier is colchicine chromosome doubling [15, 16]. Chu and Lyrene [17] provide a detailed overview of polyploid induction for *Vaccinium* interspecific hybridization in a recent review. *Vaccinium* species also tend to be self-incompatible, which facilitates easier crossing as emasculation of flowers is not always required to obtain hybrids. Non-emasculation can even aid in the success of a cross as ethelyne release is induced by emasculation and impacts fruit set negatively [18]. Nonetheless, flowers are easy to emasculate and in an intraspecific cross, pollinating one stigma can produce up to 50 viable seeds. The reasons outlined above contribute to the successes of interspecific hybridization in this group.

In this systematic review, we used a total of 64 sources, yielding 271 reported crosses between 1937 and 2023 involving 42 different *Vaccinium* species across 15 sections resulting in 136 unique species combinations (Table S1). Out of these, 53% were intrasectional and 47% were intersectional crosses. In 88% of the crosses, progeny was reported to be obtained and of those, 38% were reported at least partially fertile. Of those that reported the direction (54%), 58% performed reciprocal crosses compared to 42% one way. In addition, many crosses were between different ploidy levels (41%), and of those, colchicine for chromosome doubling was only reported to be used 11% of the time. The details and outcomes are presented below by *Vaccinium* section.

### Intrasectional hybridization

#### True blueberries (sect. *Cyanococcus*)

Blueberry is a common name used for several species. However, ‘true blueberries’ are native to North America, and belong to *Vaccinium* sect. *Cyanococcus*. The species inside this section are further split into two main categories of domesticated species: Highbush Blueberry (*V. corymbosum*), and Rabbiteye Blueberry (*Vaccinium ashei*). Wild stands of Lowbush Blueberry (*Vaccinium angustifolium*) have not been genetically improved, but they are still considered commercially valuable and are treated as a cultivated crop [19]. Taxonomically, section *Cyanococcus* is monophyletic, with all species sharing a common ancestor [1, 9, 20]. Given the short divergence periods, intermediate forms and sexual compatibility are prevalent within *Cyanococcus,* which lacks sterility barriers between homoploids [10, 21, 22]. The close relatedness and overlapping habitats of *Cyanococcus* species facilitates viability of natural and artificial hybrids. Breeders have exploited this to form a secondary gene pool in highbush blueberries.

**Figure 1:**
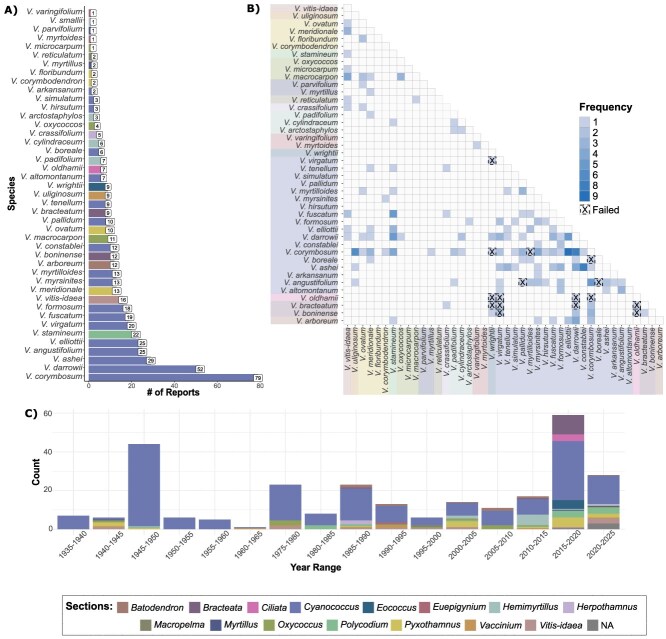
*Vaccinium* species used in interspecific crosses documented in the literature. (a) Counts of species reported as a parent*.* (b) Heatmap of the frequency of crosses between species and failure outcome. Colors in the *x-* and *y*-axes represent the taxonomic section of the species. (c) Timeline of hybridization reports across the *Vaccinium* genus. Different colors represent the sections of parents.

The domestication of highbush blueberry began with interspecific hybridization. In 1908, USDA botanist Frederick Coville and cranberry grower Elizabeth Coleman White initiated domestication attempts by crossing elite wild accessions of two tetraploid species, *V. corymbosum* and *V. angustifolium*, in New Jersey [23–25]. Coville’s early experiments also included other wild species such as *Vaccinium myrtilloides*, *Vaccinium fuscatum*, *Vaccinium pallidum*, *Vaccinium myrsinites*, and *Vaccinium hirsutum*. Since all these species belong to section *Cyanococcus*, interspecific crosses at the tetraploid level produced viable progeny [23]. Among the 15 cultivars Coville released, six were of interspecific hybrid descent: ‘Greenfield’, ‘Rancocas’, ‘June’, ‘Redskin’, ‘Catawba’, and ‘Weymouth’ [23, 24]. Including the original crosses reported by Coville, *V. angustifolium* was reported as a parent 25 times in the literature (Fig. 1a). Consequently, *V. angustifolium* is likely present in the majority of modern highbush blueberry germplasm, which have mostly started with Coville’s materials [11].

In the 1950s, Ralph Sharpe from the University of Florida furthered the hybrid nature of the northern-adapted highbush blueberry using the southern native diploid and hexaploid species, *V. darrowii* and *V. ashei,* respectively. The introgression of these low-chill species improved adaptability to warmer climates [5–7]. Initially, the strong triploid block when crossing *V. corymbosum* (4×) with *V. darrowii* (2×) posed a challenge for interploid crosses. Luckily, a *V. darrowii* wild accession (‘Fla4B’) producing a natural high frequency of unreduced gametes was discovered, facilitating the production of tetraploid hybrids. From 1600 flowers pollinated, 31 tetraploid *V. corymbosum* × *V. darrowii* hybrids were produced [6]. Additionally, a few tetraploid offspring resulted from crossing diploid *V. darrowii* with hexaploid *V. ashei* [6]. To a lesser extent, the diploid *Vaccinium tenellum* also contributed to the low-chill germplasm [26]. These pivotal hybrids led to the development of a split between northern and southern highbush blueberry germplasm. *Vaccinium darrowii* and *V. ashei* are prominently cited in literature as parents, appearing 50 and 29 times, respectively (Fig. 1a).

The horticultural community has long considered *V. ashei* and *Vaccinium virgatum* synonyms, though recent work from Fritsch *et al*. [27] found them as distinct species. Due to this common misconception, we can consider the 20 *V. virgatum* citations conflating with *V. ashei’s,* possibly increasing this group’s involvement to 49 citations (Fig. 1a).

The southeastern USA boasts a greater diversity of *Vaccinium* species compared to the northeast, offering breeders a wider range of locally adapted species for breeding. Besides *V. darrowii* and *V. ashei*, other species such as *V. fuscatum*, *Vaccinium elliottii*, *V. myrsinites*, and more distantly related species *Vaccinium arboreum* (sect. *Batodendron*) and *Vaccinium stamineum* (sect. *Polycodium*) have all been crossed with highbush blueberry (*V. corymbosum*) as depicted in Fig. 1b.

*Vaccinium elliottii* (sect. *Cyanococcus*), or Mayberry, has been notably referenced with 25 citations (Fig. 1a). Its first use was reported by Darrow and Camp in 1945, with subsequent significant contributions by the breeders Paul Lyrene and Jim Ballington. *V.accinium elliottii*, a diploid species native to the southeastern USA, is prized for its small berries with pleasant flavor, early flowering, short fruit development period, and upland soil adaptation [28]. The offspring of these interspecific hybrids resulted in cultivars ‘Carteret’ from North Carolina State University and ‘Snowchaser’ from the University of Florida [11]. Two strategies have been employed to overcome its crossing barrier into tetraploid highbush cultivars: relying on natural unreduced gamete production [29] or using colchicine to double the chromosome count [10, 30, 31]. 

Other *Cyanococcus* species used less frequently in breeding, listed by their frequency of reports in parentheses, include *V. fuscatum* (19), *Vaccinium formosum* (18), *V. myrsinites* (13), *V. myrtilloides* (13), *Vaccinium constablei* (12), *V. pallidum* (10), *V. tenellum* (9), *Vaccinium altomantanum* (7), *Vaccinium boreale* (6), *V. hirsutum* (3), *Vaccinium simulatum* (3), and *Vaccinium arkansanum* (2). This demonstrates that all species within *Cyanococcus* have been tested for breeding purposes, but not all combinations resulted in successful progenies, possibly due to limited attempts (Fig. 1b).

#### Cranberry (sect. *Oxycoccus*)

Compared to blueberries, intrasectional hybrids for cranberry improvement within sect. *Oxycoccus* have been significantly more limited. This is likely due to the fewer species in *Oxycoccus* compared to *Cyanococcus*. Three species are reported in this subgenus: *V. macrocarpon* (2*n* = 2*x* = 24), *Vaccinium oxycoccos* (2*n* = 4*x* = 48), and *Vaccinium microcarpum* (2*n* = 2*x* = 24)*,* with the last two sometimes lumped into just *V. oxycoccos* despite being two different ploidy levels [32, 33]. This taxonomy was corrected according to [34] to rename all diploid *V. oxycoccos* to *V. microcarpum*. Despite the limited gene pool in the section, cranberry breeders often use wild selections of *V. macrocarpon* and *V. microcarpum* in their breeding programs. *Vaccinium oxycoccos* in its tetraploid form has not been cited as a parent, due to incompatibility issues between diploid cranberry cultivars and a tetraploid wild species.

Cranberry was domesticated in the 1820s in Massachusetts, USA, but unlike blueberry, no breeding occurred initially—growers only planted wild selections [4]. It was not until 1929 that active breeding for cranberries began, still using only selections of *V. macrocarpon* [4]*.* Interspecific hybridization did not occur until 1975 [35].

Christ [35] reported using *V. microcarpum* (reported as diploid *V. oxycoccos* but corrected for taxonomic consistency) to introgress early blooming into cranberry. Only two more reports of *V. macrocarpon* × *V. microcarpum* hybrids have since been documented by Vorsa [26, 36]. In summary, *V. microcarpum* is cited for contributing traits such as early bloom, hardiness, and a unique profile of anthocyanins [26, 35, 36]. There have only been five references of *V. microcarpum* as a parent (Fig. 1a). In contrast to blueberry, no interspecific hybrids between species of sect. *Oxycoccus* have resulted in cranberry cultivars of commercial importance, and their applications have been limited to genetic studies. As expected, most of the hybridization involving *V. macrocarpon* are wide hybrids aiming to extend beyond sect. *Oxycoccus*.

### Intersectional wide hybridization

Wide hybridization, also defined as intersectional hybrids, are those that tap into the tertiary gene pool comprised of species that are more distantly related and from different taxonomic sections. These hybridization events, when successful, can harness greater genetic diversity across the genus.

As visualized in Fig. 1b, cross frequency clusters around sect. *Cyanococcus.* While 49% of the reported crosses are intrasectional *Cyanococcus*, 47% are intersectional over a total of 15 sections. The first reports of intersectional hybridization are from the thesis work of Schultz [37] in which taxonomic relationships were inferred from crossing experiments involving *Vaccinium* species across six sections, *Cyanococcus*, *Myrtillus*, *Oxycoccus*, *Pyxothamnus*, *Vaccinium*, and *Vitis-idaea* (Fig. 1c). Of the 128 intersectional crosses, 18 were reported as having no progeny obtained, and therefore, failed crosses. Twelve wide hybrid combinations were complete failures and have no reported success, yet all but one combination have only been tested once (Fig. 1c).

Wide hybrids are more difficult to obtain due to elusive reproductive barriers beyond ploidy level differences. Despite sharing a base chromosome number (*n* = 12) and having highly syntenic genomes across all known species [38, 39], there are still cases of cross failure with the reproductive barriers unknown. Factors influencing wide hybrid success in *Vaccinum* have been speculated to include cross direction [40], genetic variation of parental species [10], and genome balance requirements [41]. Furthermore, crosses made at the tetraploid level are more likely to be successful due to the resulting ‘allotetraploidy’ that can buffer genomic conflicts [42] and aid in regaining fertility [43].

In other plant species, the intrinsic mechanisms of hybrid failure have been attributed to prezygotic pollen–stigma incompatibility [44], and various states of postzygotic intergenomic conflicts including cytoplasmic male sterility [45] and epistatic interactions between incompatible mutations (Bateson−Dobzhansky−Muller model) such as hybrid-necrosis systems [46]. Additional causes of failure include lethal single genes [47], differences in repetitive DNA sequences such as transposable elements, and satellite DNA that can cause segregation distortion [48], as well as disruption of genomic imprinting and the misregulation of gene expression [49]. In *Vaccinium*, this remains an entirely open area of investigation that could bring new breeding tools for introgression of more distantly related species.

#### Beyond *Cyanococcus*

##### Section *Vaccinium*

The first successful reports of wide hybridization beyond the sect. *Cyanococcus* were conducted in Finland by Rousi [50], who crossed *V. corymbosum* cultivars, ‘Rancocas’ and ‘Pemberton’, with wild *Vaccinium uliginosum* individuals from the sect. *Vaccinium*. Commonly known as bog bilberry, *V. uliginosum* is a circumboreal species, occurring naturally in diploid, tetraploid, and hexaploid forms [32]. Its cold tolerance, attributed to its extreme northern distribution, has made it an attractive candidate for breeding to expand the cultivated range of highbush blueberry. The F1 hybrids from Rousi’s study were backcrossed to highbush blueberry, resulting in the cultivar ‘Aron’, which has demonstrated improved winter hardiness compared to American cultivars [51].

Since the 1960s, *V. uliginosum* has been used as a parent in nine documented hybridizations, all with members of the section *Cyanococcus* [40, 50, 52–55]. These reports are consistent, but sparse over the years (Fig. 1c).

##### Section *Polycodium*

Another important example of wide hybridization involves *V. stamineum*, commonly known as deerberry. *Vaccinium stamineum* (sect. *Polycodium*) is a diploid species with high trait polymorphism across its wide range of occurrence, from southeastern Ontario to central Florida, eastern Texas, eastern Oklahoma, and southern Kansas, with isolated populations in central Mexico. Noted for breeding by Darrow & Camp [22], it has been mentioned in 22 reports, 19 of which involve crosses with members of section *Cyanococcus* [10, 16, 22, 26, 56–58]. Interest in *V. stamineum* stems from its unique traits, including the accumulation of internal flesh pigmentation, large berry size compared to other wild species, high soluble solid content, firm texture, and adaptability to dry, sandy soils [7, 16, 59]. Following the creation of F1 hybrids, breeders backcrossed these hybrids to highbush blueberry to incorporate these traits into commercial lines.

Despite breeding efforts since 1945, it was not until 2025 when *V. stamineum* was incorporated into a cultivar released by the University of Florida. It is also noteworthy that *V. stamineum* has potential to be a standalone commercial crop [16, 58].

##### Section *Batodendron*

*Vaccinium arboreum*, commonly known as sparkleberry, from section *Batodendron* is a diploid species with a range extending from southern Virginia to central Florida, eastern Texas, central Oklahoma, and southeastern Missouri [32]. Since the 80s, *V. arboreum* has been extensively used for wide hybridization (Fig. 1c). Initially, in 1986, F1 hybrids of unknown fertility were reported between *V. arboreum* and diploid species from section *Cyanococcus* [10], but by 1991, fertile diploid F1 hybrids were successfully obtained [60]. Reports on these hybrids have continued over the years [14, 58, 61–64].

Breeders primarily value *V. arboreum* for its adaptation to challenging conditions, including dry and high-pH soils, as well as its deep taproot architecture, and floral structure that may aid in pollination [60]. *Vaccinium arboreum* represents a success story for wide hybridization, with its lineage contributing to a commercially available cultivar. The University of Florida Blueberry Breeding Program’s pedigree data show that hybrids between diploid *V. darrowii* and *V. arboreum* were backcrossed two generations with southern highbush blueberry, resulting in the cultivar ‘Meadowlark’ [65]. Additionally, F1 hybrids of ‘*V. arboreum* × highbush blueberry’ are currently tested as rootstocks to improve machine harvestability.

##### Section *Hemimyrtillus*

Species of sect. *Hemimyrtillus* including *Vaccinium arctostaphylos*, *Vaccinium cylindraceum*, *Vaccinium padilfolium*, and *Vaccinium smallii*, are native to Portuguese islands (Maderia and Azores), the Caucasus region, and Northeast Asia. These species have been of interest to blueberry breeders since the 1940s (Fig. 1c). Darrow and Camp [22] reported the first attempt at crossing *V. arctostaphylos* of sect. *Hemimyrtillus* with *V. formosum* of sect. *Cyanococcus* out of general curiosity. This cross was reported as successful, but no information on phenotype, fertility, and backcrossing was provided.

This section was not mentioned again until Ballington [26] reviewed a hybrid of diploid *V. cylindraceum* and *V. darrowii*. The progeny of this cross was described as amphidiploids and fertile. *Vaccinium cylindraceum* was especially valued for its upright growth habit, long pedicels, and loose inflorescence, which may aid in machine harvesting [26].

The most recent report of crossing species from this section with *Cyanococcus* species came from Ehlenfeldt and Ballington [57]. They outlined the crosses between *V. darrowii* and *V. cylindraceum*, *V. corymbosum* and *V. cylindraceum*, *V. angustifolium* and V*. padifolium*, *V. cormybosum* and *V. padifolium*, and a complex *Cyanococcus* hybrid and *V. smallii*. In all these crosses, progeny were obtained, but only the offspring of *V. darrowii* and *V. cylindraceum* were fertile [57].

Interestingly, Ehlenfeldt and Ballington [57] also reported a cross between *V. stamineum* (sect. *Polycodium*) and *V. cylindraceum* with no explanation, but likely with the purpose of serving as a bridge species, which are species used to overcome genetic incompatibility in wide hybridization.

##### Section *Pyxothamnus*

Section *Pyxothamnus* includes species native to the Americas as both diploid and tetraploid species. Interest in sect. *Pyxothamnus* began in the 1940s with Darrow & Camp [22] reporting hybrid seedlings between *V. darrowii* and *Vaccinium ovatum*. This interest continued sporadically over the years (Fig. 1c), with reports of crosses with *Cyanococcus* by Ballington [26], Ehlenfeldt and Ballington [66], and Ehlenfeldt and Luteyn [67].

*Vaccinium meridionale* is a tetraploid species native to high elevation regions of Peru, Colombia, and Venezuela. It has garnered considerable interest due to its reported ability to produce high-frequency triploids when crossed with diploid species of *Cyanococcus* (including *V. boreale*, *V. corymbosum*, *V. elliottii*, *V. tenellum*, and *V. darrowii*), which contrasts prior reports of a strong triploid block in *Vaccinium* [7, 66]. However, these triploids did not sustain significance for breeding purposes because of their low fertility. Because high-frequency triploid production in *Vaccinium* is so surprising, these crosses should be investigated further. A follow-up study reported the successful hybridization of *V. meridionale* × *V. corymbosum*, resulting in tetraploid offspring with both vigor and fertility [67].

Beyond its ability to produce triploid offspring, *V. meridionale* is of interest to blueberry breeders because of its monopodial plant structure and prolific concentrated flowering, both of which may aid in the development of machine-harvestable cultivars [67]. Perhaps the most promising use of *V. meridionale* is its potential to act as a bridge between taxonomic sections, expanding the potential usage of *Vaccinium* species from around the world [68]. The use of *V. meridionale* is recent, therefore, further exploration is needed to incorporate its traits into released cultivars.

##### Section *Bracteata*

Another recent development of wide hybrids has been those originating from the East Asian section *Bracteata*, including species *Vaccinium bracteatum* and *Vaccinium boninense*. The first report of *Cyanococcus* × *Bracteata* hybrids are from Tsuda *et al*. [69], which outline crossing experiments between colchicine-induced tetraploid *V. bracteatum* and *V. corymbosum* cv. ‘Spartan’. Similar to *V. stamineum*, *V. bracteatum* has internal pigmentation, a trait that was observed in the fruits of the F1 hybrids. In addition to its red pigmentation, *V. bracteatum* was targeted for its root system contributing to drought tolerance and adaptation to soils of higher pH.

The work with this section was furthered by Miyashita [70] and Miyashita *et al*. [18] with continued usage of *V. bracteatum* but an addition of *V. boninense* and *Vaccinium wrightii*. *Vaccinium wrightii,* also native to islands of East Asia, was reported as a member of sect. *Bracteata*, however, has been corrected to sect. *Eococcus* based on the phylogeny of Becker *et al*. [1]. Fertile F1 offspring of (*V. darrowii* × *V. elliottii*) × *V. boninense* was obtained and described as having light purple flesh, like previous reports. *Vaccinium wrightii* is reported to have successfully crossed with *V. darrowii* and a *V. darrowii* × *V. elliottii* hybrid but the fertility of the offspring is unknown.

The hybrids obtained with Asian sections of *Vaccinium* are promising as they are far removed from the domestication point of blueberry. This may offer a unique resource for breeders focused on the Asia/Pacific region as the regional blueberry industry has experienced significant growth in the last decade with an increase in 62 000 cultivated hectares since 2014 with China now being the top blueberry-producing country globally [71].

##### Other sections

*Vaccinium oldhamii*, belonging to sect. *Ciliata*, was another species identified as a potential parent by Miyashita [70]. Native to East Asia, *V. oldhamii* is a diploid species that also accumulates internal pigment. This trait was passed on to three F1 hybrids created with a *V. darrowii* × *V. elliottii* hybrid. However, these offspring were reported to be completely infertile.

Known as the European Bilberry, *V. myrtillus* of sect. *Myrtillus* holds cultural significance as a wild-harvested species throughout its range. It is particularly valued for its internal pigmentation, which results in a significantly higher anthocyanin content compared to blueberry cultivars [72]. Podwysynska *et al*. [73] were the first to report successful F1 hybrids of *V. myrtillus* × *V. corymbosum*. In their experiment, *V. myrtillus* underwent a colchicine treatment to induce tetraploidy before being crossed with the cultivars ‘Liberty’, ‘Bluecrop’, and ‘Northland’. While offspring were obtained for all cross combinations, fertility was not reported [73].

As the sole member of section *Herpothamnus*, *Vaccinium crassifolium*, known as creeping blueberry, is a diploid species endemic to four southeastern states in the USA [32]. A review by Lyrene and Ballington [10] listed a hybrid between *V. tenellum* of sect. *Cyanococcus* and *V. crassifolium* with partial fertility. The second, and last, report of a *V. crassifolium* hybrid with *Cyanococcus* comes from Redpath *et al*. [74], who simply reported the genome size of a *V. fuscatum* × *V. crassifolium* hybrid available in the National Clonal Germplasm Repository (NCGR). The minimal reports on *V. crassifolium* usage, coupled with the absence of published discussions about its breeding potential, limited its true value to improving cultivated germplasm.

Another sole member of its section is *Vaccinium vitis-idaea*, also known as lingonberry, of section *Vitis-idaea*. *Vaccinium vitis-idaea* is a diploid circumboreal species that is culturally significant in Scandinavia [32, 75]. A *V. darrowii* × *V. vitis-idaea* hybrid was the first *Cyanococcus* × *Vitis-idaea* cross reported by Ballington [26] as a personal communication from Nicholi Vorsa. Following this report, Ehlenfeldt *et al*. [41] reported two crosses: *V. vitis-idaea* × *V. fuscatum* and *V. vitis-idaea* × *V. elliottii* with only the former resulting in offspring that were strictly male fertile. Interestingly, though both *V. vitis-idaea* and *V. fuscatum* are diploid, the offspring were reported as triploid. Furthermore, the triploid offspring were able to cross-pollinate with 6x *V. virgatum*, indicating the production of unreduced gametes in the F1.

#### Beyond *Oxycoccus*

##### Section *Vitis-idaea*

As previously discussed, section *Oxycoccus* has only three species: *V. macrocarpon*, *V. oxycoccos*, and *V. microcarpum*. Consequently, intersectional hybridization has been suggested as a method to genetically improve cranberries and introgress novel traits, though its contributions are not yet commercially significant. The earliest reports of breeding with species of sect. *Oxycoccus* involved an intersectional hybrid between *V. macrocarpon* and *V. vitis-idaea* to introgress its unique aroma [35, 76]. Following the 1970s, this hybrid was no longer reported. Additionally, Schultz [37] reported success in obtaining progeny between these two species.

##### Section Macropelma

In the late 1990s, Zeldin and McCown [77] explored the use of *Vaccinium reticulatum*, a species native to Hawaii within section *Macropelma* to generate hybrids with *V. macrocarpon* and *V. vitis-idaea* (Fig. 1c). The main goal was to broaden the genetic basis of cranberry and lingonberry [77]. Traits of interest in *V. reticulatum* included its large red fruit, pest resistance, higher freeze tolerance, drought tolerance, and the ability to develop flowers on new growth, eliminating the need to protect buds during overwintering. While progeny was obtained, very little pollen was shed from the hybrids with *V. macrocarpon*. However, the crosses with *V. vitis-idaea* successfully set fruit, and F2 seedlings were obtained. No subsequent reports have documented further crossing involving members of sect. *Macropelma*.

##### Section *Pyxothamnus*

Species of sect. *Pyxothamnus* have been instrumental in expanding the cranberry gene pool as they have been considered a bridge for intersectional hybridization [68]. *Vaccinium meridionale* has been an especially promising resource as it crosses with distant related species of sect. *Cyanococcus*, *Oxycoccus*, and *Vitis-idaea* [43, 66, 67]. F1 hybrids of tetraploid *V. meridionale × V. macrocarpon* are described as intermediate with limited female fertility and good male fertility. The authors suggest that the offspring is allotetraploid and has potential to generate an upright cranberry-like cultivar and expand the range of production [41].

##### Section *Cyanococcus*

Finally, a single report of a *Cyanococcus* × *Oxycoccus* hybrid was reported by Vorsa *et al.* [78] at the diploid level. This cross occurred between *V. darrowii* × (*V. macrocarpon* × *V. microcarpum*). Despite this accomplishment, the offspring were infertile. Ehlenfeldt *et al*. [43] suggested that these crosses are more likely to result in fertile offspring when conducted at a tetraploid level so that the resulting allotetraploidy can aid in reinforcing fertility.

### Considerations

Relying solely on historical crossing reports for understanding the germplasm base presents several challenges including taxonomic changes, species misidentifications, and missing crossing records. Changes in taxonomy pose a significant challenge when sorting through crossing records since names have frequently changed over time, especially within sect. *Cyanococcus*. To improve the robustness of future interspecific hybridization studies, breeders and researchers should consider creating and citing herbarium specimens of the parental species. Furthermore, our review can only include hybridizations that have been reported in the literature. Many hybrids may have gone unreported. Most reports highlight success stories, while failures often remain unpublished. This bias is reflected in our statistics, as 88% of all reports claim progeny was obtained from the cross, thus inflating the success rate of interspecific hybridization. Despite these challenges, the integration of genomic technology offers a way to infer the contribution of wild species into the current genetic background of cultivars.

## Vaccinium diversity and germplasm resources

### Defining the gene pool

The crop gene pools are typically split into three categories: primary, secondary, and tertiary gene pools. These gene pools help to define crossability among crop wild relatives. For blueberry, Lyrene and Ballington [10] define the primary gene pool as wild individuals of *V. corymbosum or V. ashei* for highbush and rabbiteye, respectively, the secondary genepool as the rest of *Cyanococcus* species, and the tertiary being the rest of *Vaccinium*. Similarly, for cranberry, the primary gene pool is *V. macrocarpon*, the secondary gene pool is other members of *Oxycoccus* such as *V. oxycoccos* and *V. microcarpum*, and the tertiary gene pool is the rest of *Vaccinium* [26].

**Figure 2:**
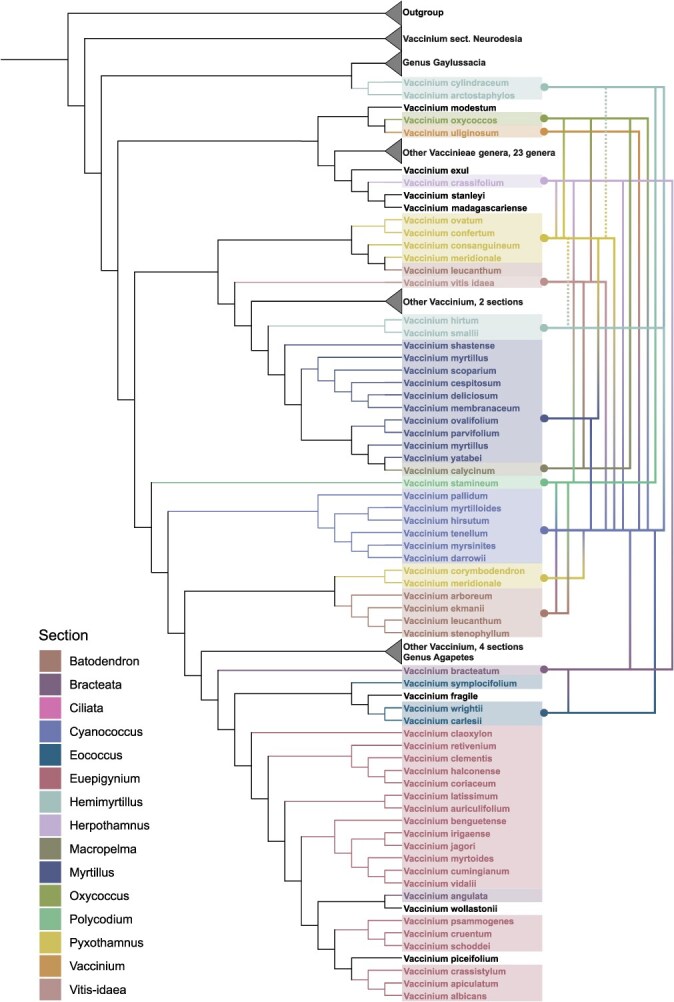
Phylogeny of the *Vaccinium* genus highlighting species and sections used for hybridization experiments. Crosses between sections are indicated by colored brackets. Dashed lines indicate a polyphyletic section with ambiguous phylogenetic origin. Figure was adapted from Becker *et al*. [1].

While prior work has defined the extent of the gene pool for blueberry and cranberry to the genus *Vaccinium* [26], we argue that the known polyphyly of *Vaccinium* demands consideration of the entire tribe when describing the wild relatives of blueberry and cranberry. Becker *et al*. [1] greatly expanded the phylogenetic analyses of the tribe Vaccinieae by increasing species sampling (261 terminals) and loci analyzed (256 low-copy nuclear genes from the Angiosperm353 v1 target capture kit). Utilizing this most recent phylogenic tree topology from Becker *et al*. [1] and the historical records of hybridizations compiled in this study, we can redefine potential crop wild relatives for these crops (Fig. 2).

Acknowledging the near-complete cross-compatibility between homoploids of sect. *Cyanococcus*, we can consider this monophyletic group as one entity for crossability. *Cyanococcus* has been demonstrated to successfully cross with species of 12 sections: *Hemimyrtillus*, *Herpothamnus*, *Oxycoccus*, *Vaccinium*, *Pyxothamnus*, *Vitis-idaea*, *Myrtillus*, *Bracteata*, *Eococcus*, *Ciliata*, *Batodendron*, and *Polycodium*. All sections are represented on the phylogenic tree (Fig. 2), except sect. *Ciliata*. The phylogenetic distance of many of these sections from *Cyanococcus* have striking implications for the possibilities of wide hybridizations. However, perhaps more interesting than the distance of these *Vaccinium* sections is the presence of the collapsed clade containing 23 other genera in between *Vaccinium* species. *Cyanococcus* has demonstrated its ability to cross to species on either side of this collapsed clade, indicating the potential to cross with species currently classified as other genera. These genera are mostly distributed in South and Central America, with just *Dimorphanthera* distributed throughout Southeast Asia. Furthermore, the documented interspecific hybrid of *Vaccinium* × *Agapetes* found in the USDA GRIN database is an example of an intergeneric hybrid that may be indicative of greater diversity available for breeding than previously thought [79].

### Species richness and repositories

First defined by Linneaus in 1737, the genus *Vaccinium* is often reported as comprising ~500 species within the subfamily Vaccinoideae and tribe Vaccineae which contains ~34 genera and ~1430 species [1]. *Vaccinium* is a cosmopolitan genus with a near global distribution, but the greatest density of species are found in the tropics [21]. According to current biodiversity databases, the species richness of *Vaccinium* is highest in Southeast Asia, specifically Indonesia, followed by either Papua New Guinea or China depending on the database (Fig. S1).

**Figure 3:**
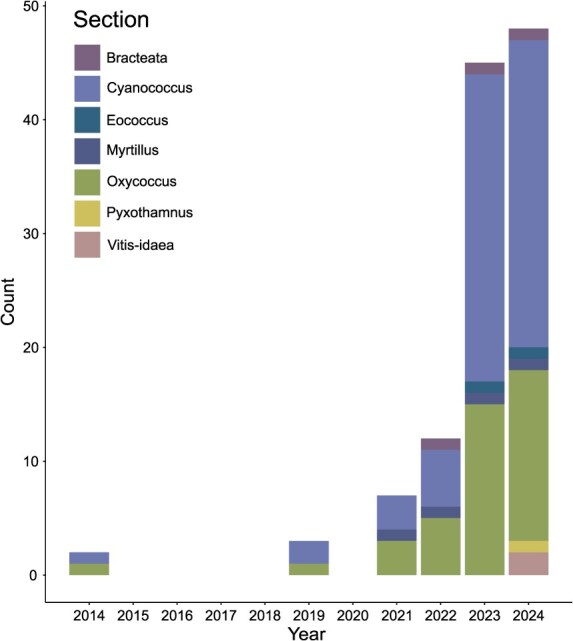
Cumulative increase in the number of publicly available genome assemblies for *Vaccinium* species over the years. Different taxonomic sections within *Vaccinium* are labeled by color.

While it is agreed that *Vaccinium* is a large genus with vast diversity, the true number of species and their distributions are difficult to pinpoint. The two standard biodiversity databases, Global Biodiversity Information Facility (GBIF) and Integrated Digitized Biocollections (iDigBio), are inconsistent for species reports, synonyms, and richness by country (Fig. S1). Database comparisons can be found in File S1. These biodiversity databases and herbarium records are a severely underutilized resource for plant breeders and can play a valuable role in determining germplasm collection goals. More efforts and funds should be dedicated to curate the information and expand accessions for underrepresented taxa and countries. In this sense, it is also important to acknowledge the role of the USDA National Clonal Germplasm Repository (Corvallis, OR, USA). In their Germplasm Resource Information Network (GRIN), there are 76 species across 1841 accessions within the genus *Vaccinium* available upon request.

### Genomic resources

In the era of genomics-assisted breeding, generating DNA sequence information for wild species can also aid in speeding up prebreeding efforts. Although diverse and well adapted to their native environment, wild species often possess several undesirable traits and linkage drags can be difficult to overcome. The precise genomic identification of major domestication loci in cultivated species or regions controlling traits of interest to introgress from the wild species have been shown in other crops to accelerate the recovery of the elite background [80] and/or for the *de novo* domestication of wild species [81]. In blueberry, researchers have used genomics to uncover historical introgressions from wild species. For instance, Brevis *et al*. [82] and Nishiyama *et al*. [83] used molecular markers to uncover the admixture of various *Vaccinium* species in highbush and rabbiteye blueberry cultivars. Similarly, Wang *et al*. [84] conducted genomic analysis to identify genes associated with low-chilling requirements and fruit firmness introgressed from *V. darrowii* and V*. ashei* into southern highbush blueberries.

The first draft genomes of blueberry [85] and cranberry [86] were published in 2014. But a high-quality and chromosome-scale assembly of blueberry only became available in 2019 [87] and for cranberry [88] in 2021. Since then, advancements in sequencing technology and bioinformatic tools promoted a rise in the number of genome sequences available, with some tapping into wild species of undomesticated sections (Fig. 3, Table S1). Available genomes of wild species include*, V. darrowii* [89, 90], *Vaccinium caesariense* [91], *V. microcarpum* [88, 92], *V. myrtillus* [39], *V. vitis-idaea* [93], *V. bracteatum* [94], *Vaccinium duclouxii* [95], and *Vaccinium floribundum* [96]. A highbush blueberry and cranberry pangenome became recently available but species outside of sect. *Cyanococcus* and *Oxycoccus* are not included, therefore only a subset of *Vaccinium* diversity is represented [97]. While other crops already have super-pangenomes in place [98], the diversity of the *Vaccinium* genus is still tremendously underrepresented in terms of genomic resources.

### Global need for prebreeding efforts

Although we have demonstrated the potential for crossability with other *Vaccinium* species and genera of the tribe Vaccinieae, crucial information about these wild species is still missing, which is imperative for effective prebreeding. While close relatives of blueberry and cranberry, such as those in sections *Cyanococcus* and *Oxycoccus*, are well documented, most sections remain poorly described. A simple record search on iDigBio (iDigBio) reveals that information on most tropical *Vaccinium* species is limited to <10 records (Fig. S1), posing a significant limitation for prebreeding collections.

Despite *Vaccinium’s* near-global distribution, most breeding work and reports of interspecific hybridization are occurring in the USA, where both blueberry and cranberry are native and have been domesticated. However, due to the global expansion of production areas, the use of new species may be crucial for the local adaptation and improvement of these crops [3]. China (in Asia) and Peru (in South America) have already surpassed the USA in terms of blueberry production [99]. The successful adaptation of southern highbush cultivars to the warm climate in Florida using the native *V. darrowii* [7] and to harsh Scandinavian winters using the native *V. uliginosum* [51] are examples of the possibilities when developing cultivars for new regions.

Within the USA, hybridization experiments have made notable efforts to harness global diversity for breeding, with reports including parent species from 14 sections, whereas other countries’ publications have never exceeded the use of species from five sections. Countries with the highest species richness, such as Indonesia, Papua New Guinea, and China, have not published any work on interspecific hybridization involving their native species (Fig. S1). This disparity could be due to the English-query bias of this study, but also due to a lack of publications, resources, or interest in these other countries. The majority of cross reports were made by five US-based breeders: Dr Paul Lyrene, Dr Mark Ehlenfeldt, Dr James Ballington, Dr Nicholi Vorsa, and Dr James Polashock. An overview of authorship patterns can be found in File S1 and Fig. S2.

## Perspectives

Overall, this review reinforces the need for a more globalized effort to characterize local native germplasm resources and report hybridization outcomes (both successes and failures) with *Vaccinium* species. Locally adapted cultivars have proven themselves imperative by the examples outlined in this review and should continue to be generated for each growing region. Furthermore, the near-global distribution of *Vaccinium* and the opportunities outlined by phylogenetic analyses highlight the unique case of blueberry and cranberry as relatively novel crops moving into the global sphere. With a unified, worldwide effort in *Vaccinium* prebreeding, the gaps in our understanding of species distributions, characteristics, and crossability can be bridged, paving the way for a more sustainable and resilient future for these increasingly valuable and health-promoting crops.

## Supplementary Material

Web_Material_uhaf246
