# Supplementary material for: Interspecific hybridization history of Vaccinium berry crops and potential in wild relatives
Source: Hortic Res. 2025 Sep 17;12(12):uhaf246. doi: 10.1093/hr/uhaf246 (PMC12703620; doi:10.1093/hr/uhaf246)
Supplement: Web_Material_uhaf246 [file web_material_uhaf246.zip › Sup_File1_fixed.docx]

Interspecific Hybridization History of *Vaccinium* Berry Crops and Potential in Wild Relatives

**Authors:** Anisa M. Khalid^1^, Juliana Benevenuto^1^, Paul M. Lyrene^1^, Patricio R. Munoz^1^*

Affiliation: 1 Horticultural Sciences Department, University of Florida, Gainesville, FL 32611, USA

**1 Methods**

Our literature review methodology comprised of two main parts: 1) systematic screening of the literature and 2) independently finding historical records through databases, books, and other sources. We conducted a systematic literature search using the ISI Web of Science Core Collection (all editions) as the primary database with the search terms ((ALL=(blueberry)) OR ALL=(vaccinium)) AND (ALL=(hybrid*)) OR ALL=(interspecific)). The search was finalized on 20 February 2024 and yielded 555 potential studies. Our initial screening ruled out studies that did not focus on artificial *Vaccinium* hybridization based on the title and abstract and narrowed our list of studies down to 88. The full text of the remaining studies went through a second round of screening to remove studies that were meeting abstracts with a narrow presentation of results or because they were redundantly describing further experimentation on a hybrid that was previously reported and cited by the same author in a paper. We aimed to retrieve just the raw number of reported hybrids rather than reporting on the hybrids described. After this secondary screening process, 42 studies remained. An additional 19 studies were included and found from references of the screened studies and historical records. This screening process resulted in 62 total studies to be included in our report. Lastly, we included reported hybrids found in the United States Department of Agriculture Germplasm Resources Information Network (USDA-GRIN) online database. We concluded with 64 total sources included in our review.

Full texts of the total studies were screened to report species crossed, their sections, their ploidies, the direction of the cross (one way or reciprocal), whether progeny was obtained, whether this progeny was fertile, the number of seedlings per flower pollinated, whether colchicine was used to aid in the cross, and the ploidy of the progeny. In some cases, seedlings per flower pollinated were manually calculated using the data reported in the study. Special notes on the results of the studies were also included in our data collection process. Additionally, we reported on the year, authors, title, journal, and country for each of the studies. The complete summary table is available in Sup. Table 1.

Historical records with names that have been revised were corrected using updated taxonomy (Peter Fritsch, pers. comm.). Notes of the originally reported names is available as well as the corrected names in Sup. Table 1. This species name revision process mostly occurred for sect. *Cyanococcus* as it has been the most impacted by taxonomic revisions throughout history. The only official treatment currently available of *Vaccinium* sect. *Cyanococcus* is widely disputed by botanists due to rampant lumping, especially into *V. corymbosum*, therefore, to avoid nullifying most of the crosses, we renamed the recently resurrected taxa while official treatments are in progress (Franck & Salman, 2024; Fritsch et al., 2024; Vander Kloet, 1988; Weakley et al., 2024). The name revisions resulted in one null interspecific hybrid: *V. lamarckii* x *V. britonnii* to *V. angustifolium* x *V. angustifolium*. We also corrected all diploid V. oxy

To highlight the diversity of the *Vaccinium* genus globally, we downloaded species occurrence data from Global Biodiversity Information Facility (GBIF) and Integrated Digitized Biocollections (iDigBio). Occurrence data was cleaned to remove hybrids, subspecies, and occurrences missing location. Species richness by country for each databases was calculated and visualized using the *ggplot2* R package (Wickham, 2016).

The most recent phylogeny of Vaccinineae’ tribe was obtained from Becker et al. (2024) with the corresponding author’s permission. The phylogenic tree topology file (.tre) was visualized and edited using the Interactive Tree of Life v5 (iTOL) (Letunic & Bork, 2021). Revisions included refining the outgroup to contain a single clade, collapsing clades that were composed of species, sections, or genera not included in the list of hybrids reported in this study, and removal of duplicate samples for the same species that formed a clade.

**2 Supplementary Results**

2. 1 Database Comparisons

Two major biodiversity databases, GBIF and iDigBio were used to compare available information on Vaccinium species Striking inconsistencies between the databases were found. These databases report species richness for country differently, likely due to outdated taxonomy. Global species richness comparisons across these databases can be visualized in Sup. Fig. 1a. While general patterns remain the same (ex. Asia having the highest richness and Africa the lowest), differences in reports between the countries is apparent.

Reported species were inconsistent as iDigBio includes records for 470 species and GBIF only 362. There is an overlap of 299 species between the two databases but combined there are 533 unique species names reported (Sup. Fig. 1b). We also observed extreme unbalances across species regarding the number of herbarium specimen records per species (Sup. Fig. 1c). Between both databases, 71% of species have less than 100 records and 18% have less than 10. The species with the top 10 records for both GBIF and iDigBio are native to North America and Europe, with the exception of *V. bracteatum* and *V. floribundum*, native to East Asia and South America respectively. This highlights a bias in available information for global *Vaccinium* resources and an overrepresentation of species native to North America and Europe.

**Sup. Fig. 1.** Comparison of biodiversity databases GBIF and iDigBio. ***a.*** *Vaccinium* species richness per country according to both databases, ordered by continent from highest to lowest *Vaccinium* species richness. ***b.*** Venn Diagram of recognized species in both databases with common species in the center and unique species to each database on the sides. ***c.*** Herbarium specimen records available in both databases of Vaccinium species.

2.2 Authorship Patterns

Of the 64 sources analyzed, more than half (56.2%) were authored by at least one of the top five contributors: Paul Lyrene, Mark Ehlenfeldt, James Ballington, Nicholi Vorsa, and James Polashock (Sup. Fig. 2). Notably, these five researchers are the only ones with at least five publications, highlighting their dominant role in shaping the current understanding of Vaccinium interspecific hybridization. This concentration of authorship underscores a significant imbalance in the literature. However, their contributions have been instrumental in advancing Vaccinium breeding, and without their work, the field’s knowledge base would be considerably limited. Their collective expertise has laid the foundation for *Vaccinium* breeding research, emphasizing both the need to diversify contributions and the importance of building upon their work to further advance the field.

**Sup. Fig. 2.** Contribution of different breeders to Vaccinium interspecific hybridization efforts. The left pie chart represents the proportion of studies that include a top five author. The right pie chart provides a detailed breakdown of the contributions of the top five individual breeders.

Becker, A. L., Crowl, A. A., Luteyn, J. L., Chanderbali, A. S., Judd, W. S., Manos, P. S., Soltis, D. E., Smith, S. A., Goncalves, D. J. P., Dick, C. W., Weaver, W. N., Soltis, P. S., Cellinese, N., & Fritsch, P. W. (2024). A global blueberry phylogeny: Evolution, diversification, and biogeography of Vaccinieae (Ericaceae). *Molecular Phylogenetics and Evolution*, *201*, 108202. https://doi.org/10.1016/j.ympev.2024.108202

Franck, A. R., & Salman, D. (2024). *Scientific Note: Typification of Vaccinium elliottii (Ericaceae), a Distinct Species*. *89*.

Fritsch, P. W., Crowl, A. A., Ashrafi, H., & Manos, P. S. (2024). Systematics and Evolution of Vaccinium Sect. Cyanococcus (Ericaceae): Progress and Prospects. *Rhodora*, *124*(998–9), 301–332. https://doi.org/10.3119/22-10

Letunic, I., & Bork, P. (2021). Interactive Tree Of Life (iTOL) v5: An online tool for phylogenetic tree display and annotation. *Nucleic Acids Research*, *49*(W1), W293–W296. https://doi.org/10.1093/nar/gkab301

Vander Kloet, S. P. (1988). *The Genus Vaccinium in North America*. Research Branch, Agriculture Canada. https://books.google.com/books?id=pLCXzQEACAAJ

Weakley, A. S., LeBlond, R. J., McMillan, P. D., Sorrie, B. A., Poindexter, D. B., Fuller, J. B., Bridges, E. L., Budach, B. J., Carr, S. C., Crowl, A. A., Manos, P. S., Fritsch, P. W., Orzell, S. L., Wipff, J. K., Messec, L. A., Dellinger, B., Ungberg, E. A., Yawn, N. D., Cressler, A. M., … Mears, R. L. (2024). Studies in the vascular flora of the southeastern United States. X. *Journal of the Botanical Research Institute of Texas*, *18*(1), 17–77. https://doi.org/10.17348/jbrit.v18.i1.1338

Wickham, H. (2016). *ggplot2: Elegant Graphics for Data Analysis* (2nd ed. 2016). Springer International Publishing : Imprint: Springer. https://doi.org/10.1007/978-3-319-24277-4
